# Comparative analysis of conventional vs high-resolution anorectal manometry methods

**DOI:** 10.1371/journal.pone.0333188

**Published:** 2025-10-09

**Authors:** Silvana Marques e Silva, Marcelo de Melo Andrade Coura, Romulo Medeiros de Almeida, Isabel Ferreira Saenger, Valéria Cardoso Pinto, João Batista de Sousa

**Affiliations:** 1 Medical Sciences Postgraduate Program, University of Brasília, Brasília, Federal District, Brazil; 2 Federal District Health Department, Coloproctology Service, Sobradinho Regional Hospital, Brasília, Federal District, Brazil; 3 Faculty of Medicine, University of Brasília, Brasília, Federal District, Brazil; 4 Federal District Health Department, Coloproctology Unit, Gama Regional Hospital, Brasília, Federal District, Brazil; Ladoke Akintola University of Technology Teaching Hospital: LAUTECH Teaching Hospital, NIGERIA

## Abstract

**Introduction:**

High-resolution anorectal manometry (HR-ARM) is gradually replacing conventional anorectal manometry (C-ARM). Despite reports of a strong correlation between the two methods in pressure measurements, normative values vary significantly across different devices. This study, therefore, compares manometric parameters between C-ARM and HR-ARM performed in the same individuals.

**Materials and methods:**

Fifty consecutive symptomatic patients requiring anorectal manometry, irrespective of their primary complaint, underwent both C-ARM and HR-ARM performed by the same examiner. Agreement between the two methods was assessed based on the type of variable: the intraclass correlation coefficient was used for continuous parameters, while simple or weighted Kappa coefficients were used for categorical parameters.

**Results:**

The study cohort (n = 50) had a mean age of 58.06 years, and 78% were female. For continuous variables, agreement between C-ARM and HR-ARM was excellent for resting and squeeze pressures but poor for functional anal canal length. When these pressures were categorized based on each method’s normative values, the diagnostic agreement for anal tone was only fair to moderate. Regarding specific disorders, the agreement for dyssynergia markers was moderate. However, it was only fair for rectal relaxation and poor for intra-rectal propulsion.

**Conclusions:**

C-ARM and HR-ARM show excellent agreement for the quantitative assessment of resting and squeeze pressures. However, when these pressures are categorized using currently available normative values, the diagnostic agreement between the methods is poor. This strongly suggests that unique, device-specific reference ranges must be established to ensure accurate clinical interpretation.

## Introduction

Anorectal manometry (ARM) is a complementary method for diagnosing anorectal dysfunctions by evaluating the complex motor-sensory mechanisms of continence and defecation [1,2]. When combined with other anorectal function tests, neurophysiological techniques, and ultrasound, ARM provides a more comprehensive assessment of continence and evacuation disorders [3].

ARM can be performed with several types of catheters, including solid-state (SS), water-perfused (WP), and air-perfused systems [4]. In Brazil, the WP catheter is the most prevalent method, largely due to its lower cost compared to SS microtransducers.

Conventional manometry (C-ARM), which typically uses 8 radial channels, is gradually being replaced by high-resolution anorectal manometry (HR-ARM). This newer technology offers significant advantages, providing a continuous and dynamic spatiotemporal map of anorectal pressures and thereby facilitating a more detailed and straightforward data interpretation [4].

While some studies report a good correlation in pressure measurements between C-ARM and HR-ARM, they also find that high-resolution (HR) probes yield significantly higher resting and squeeze pressures [5–7]. This underscores a broader issue: normative values vary significantly among manometry devices, necessitating validation for each specific type of equipment. This problem is particularly relevant in Brazil, where no standardized normative data for HR-ARM are available. Consequently, clinicians must rely on reference values from American and European studies that were conducted using different technologies.

The primary objective of this study is to compare manometric parameters between C-ARM and HR-ARM within the same patient cohort, irrespective of the clinical indication for testing.

## Materials and methods

This prospective study was approved by the Research Ethics Committee of Unieuro University Center (report # 6.523.955). Consecutive symptomatic patients with indications for ARM were enrolled, irrespective of their primary complaint, from December 1, 2023, to March 15, 2024.

All patients signed an informed consent form and completed the Bristol Stool Form Scale. Additionally, constipated patients completed the Constipation Severity Index questionnaire [8], and incontinent patients completed the Jorge-Wexner Incontinence Score questionnaire [9].

All examinations were performed by the same examiner with the patient in the left lateral decubitus position and without prior bowel preparation. The order in which each patient underwent C-ARM and HR-ARM was determined one day in advance via simple randomization using Microsoft Excel®.

**Fig 1:**
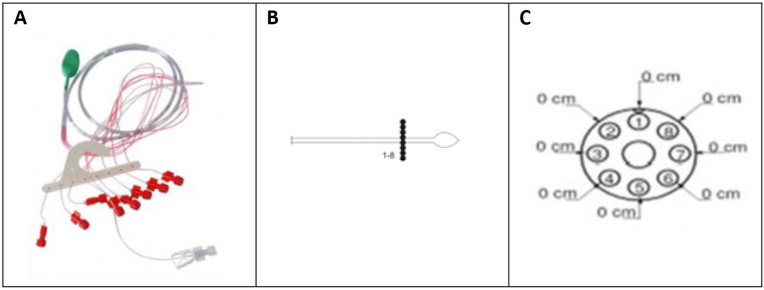
Conventional anorectal manometry. **(A)** Probe **(B)** 8 -Radial channels **(C)** Front view.

For the C-ARM method, a 0.5 cm 8-channel WP catheter (Dymaned® Dynapac MPX816) was used (Fig 1). The catheter was first inserted until its tip was positioned 6 cm proximal to the anal margin. To measure resting and squeeze pressures, a station pull-through technique was employed. The patient was instructed to first relax and then squeeze the anal sphincters. This rest-squeeze sequence was repeated as the catheter was withdrawn in 1 cm increments until it reached the anal margin (pull through technique). Following the pressure measurements, the rectoanal inhibitory reflex (RAIR) was assessed. For this test, the catheter was positioned with the balloon in the rectum and the pressure sensors in the high-pressure zone of the anal canal. The balloon was then rapidly inflated with 20–60 ml of air to assess for sphincter relaxation. Finally, two additional maneuvers were performed: anal pressure was measured during a Valsalva maneuver (with the balloon deflated) and external anal sphincter fatigue was assessed after a 30-second sustained voluntary contraction.

### From: Dynamed®/Alacer ®

Considered normal values for C-ARM are described in Table 1.

**Table 1:** Normal values for conventional anorectal manometry.

Parameter	Female		Male	
	Minimum	Maximum	Minimum	Maximum
**Functional anal canal length (cm)**	2 cm	3 cm	2.5 cm	3.5 cm
**Anal resting pressure (mmHg)**	40 mmHg	70 mmHg	40 mmHg	70 mmHg
**Anal squeeze pressure (mmHg)**	100 mmHg	180 mmHg	100 mmHg	180 mmHg
**Long (endurance) squeeze**	present	present	present	present
**First sensation volume (ml)**	10 ml	40 ml	10 ml	40 ml
**Maximal tolerated volume (ml)**	100 ml	300 ml	100 ml	300 ml
**Rectoanal inhibitory reflex**	present	present	present	present
**Voluntary relaxation**	present	present	present	present

### From: Jorge (1993) [9]

**Fig 2:**
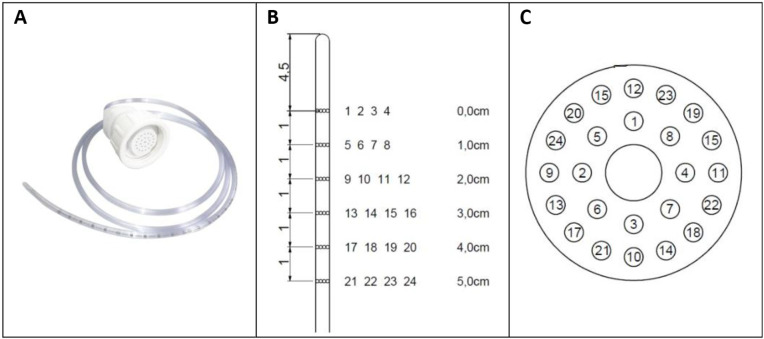
High-resolution anorectal manometry. **(A)** Probe **(B)** 24- Channels **(C)** Back view.

For the HR-ARM method, a 24-channel WP system (Alacer®, Alacer Biomédica, São Paulo, Brazil) was used (Fig 2). This system featured a continuous capillary perfusion unit controlled by a peristaltic pump, which maintained a constant water flow of 0.6 ml/min to a series of piezoelectric pressure sensors. The probe itself was constructed from polyvinyl chloride and had a diameter of 4.7 mm. Its 24 pressure channels were configured in six groups of four, with the channels in each group arranged radially at 90-degree intervals. These groups were spaced0.8 cm apart along the length of the catheter. A separate central channel connected to a latex balloon at the probe’s distal tip.

### From: Alacer ®

All HR-ARM examinations were performed in accordance with the London Protocol [10]. Following catheter insertion and prior to any test maneuvers, a 3-minute stabilization period was allowed for the anal tone to return to baseline. Resting pressure was then recorded over a 60-second interval, during which the patient was instructed to remain relaxed and quiet to avoid movement artifacts. Subsequently, squeeze pressure was measured during three consecutive 5-second anal contractions, separated by 30-second recovery intervals. For the final analysis, the attempt yielding the highest pressure was selected. First, an endurance squeeze test was performed to assess sphincter fatigue. This involved a single, sustained voluntary contraction for 30 seconds, which was followed by a 60-second recovery period. Next, simulated defecation was evaluated by having the patient perform three 15-second pushes with the catheter’s balloon deflated. These attempts were separated by 30-second rest intervals. For the analysis of this maneuver, the attempt deemed most qualitatively normal was selected. A single RAIR was performed with a starting volume of at least 20 ml.

The HR-ARM software defines the functional anal canal length (FACL) using two distinct methods:

1. The length of the canal where pressure is greater than 50% of the maximum resting pressure (HR-ARM-FACL 50%).
2. The region where pressure is at least 10 mmHg higher than the intra-rectal pressure (HR-ARM-FACL 10 mmHg).

Because there is currently no consensus on which definition is more clinically relevant, we conducted our analysis using both methods.

Considered normal pressure values in HR-ARM are shown in Table 2.

**Table 2:** Normal values for high-resolution anorectal manometry.

Parameter	Female		Male	
	Minimum	Maximum	Minimum	Maximum
**Functional anal canal length (cm)**	2.3	4.5	2.9	4.8
**Anal resting pressure (mmHg)**	44	110	54	111
**Anal squeeze pressure (mmHg)**	51	194	90	328
**Total anal squeeze pressure (mmHg)**	95	304	144	439
**Long (endurance) squeeze (%)**	≥ 50% of initial squeeze		≥ 50% of initial squeeze	
**Rectal propulsion in simulated evacuation**	≥ 40 mmHg		≥ 40 mmHg	
**Anal relaxation in simulated evacuation**	≥ 20% of resting basal pressure		≥ 20% of resting basal pressure	
**First sensation volume (ml)**	20	58	20	72
**Maximal tolerated volume (ml)**	60	140	60	212
**Rectoanal inhibitory reflex**	≥ 25% of resting basal pressure		≥ 25% of resting basal pressure	

Adapted from: E V Carrington et al. Neurogastroenterol Motil. 2020 [10] and N R Oblizajek et al. Neurogastroenterol Motil. 2019 [11].

To ensure an independent assessment, the C-ARM analysis was performed first. The HR-ARM analysis was conducted one week later by the same examiner, who did not have access to the results or calculated data from the prior C-ARM analysis.

All statistical analyses were performed using SAS software, version 9.4 (SAS Institute Inc., Cary, NC, USA). To assess agreement between C-ARM and HR-ARM for continuous variables (anal resting pressure, anal squeeze pressure, and functional anal canal length), the intraclass correlation coefficient (ICC) was calculated. We used a two-way random-effects model based on absolute agreement [12]. The resulting ICC values were interpreted according to the guidelines described by Fleiss [13]: values <0.40 indicated poor agreement; 0.40–0.59, fair agreement; 0.60–0.74, good agreement; and ≥0.75, excellent agreement. All ICCs are reported with their 95% confidence intervals (CI).

Agreement for categorical variables was assessed using the simple Kappa coefficient (K). These variables included resting and squeeze tone classifications (normotonic, hypotonic, etc.), intra-rectal propulsion, rectal relaxation, and visual signs of dyssynergic defecation. For this analysis, the continuous functional anal canal length (FACL) data was converted into a binary categorical variable (“normal” vs. “altered”). A necessary exclusion was made for the anal tone analysis. Because no patients were classified as hypertonic by HR-ARM, patients classified as hypertonic by C-ARM were excluded from this specific Kappa calculation to ensure a valid comparison. For the single ordinal variable—the position of the highest-pressure zone—agreement was assessed using the weighted Kappa coefficient (Kw) to properly account for the ordered nature of the categories. Both Kappa (K) and weighted Kappa (Kw) values were interpreted according to the Landis and Koch scale: 0.00–0.20 indicated slight agreement; 0.21–0.40, fair; 0.41–0.60, moderate; 0.61–0.80, substantial; and >0.80, almost perfect agreement [13]. The Bland-Altman plot was used for assessing the magnitude of disagreement (including systematic differences), spotting outliers, and identifying any potential trends.

## Results

A total of 51 patients were initially enrolled in the study. One patient was subsequently excluded due to a previous proctocolectomy with an ileal reservoir, leaving a final cohort of 50 patients for analysis.

The mean age of the patient cohort was 58.06 ± 11.73 years (range: 31–78), and 78% of participants were female. Regarding obstetric history, the mean number of gestations was 2.69 ± 1.64, and the mean number of vaginal deliveries was 1.51 ± 1.55. Two patients had more than one indication for ARM, and one patient had two previous anorectal surgeries. Complete clinical and demographic characteristics are provided in Table 3.

**Table 3:** Demographic and clinical characteristics of patients.

Variable	Frequency (N = 50)	Percentage (%)
**First exam**		
**Conventional anorectal manometry**	26	52
**High-resolution anorectal manometry**	24	48
**Gender**		
**Female**	39	78
**Male**	11	22
**Indication**		
**Constipation**	16	32
**Fecal incontinence**	13	26
**Hemorrhoids**	10	20
**Anorectal fístula**	10	20
**Colostomy/ileostomy closure**	3	6
**Previous surgeries**		
**None**	32	64
**Hemorrhoids**	8	16
**Abscess drainage/ Fournier**	5	10
**Fistulotomy**	3	6
**Sphincterotomy**	2	4
**Rubber band ligation**	1	2
**Frequency of bowel movements**		
**Up to every 3 days**	46	92
**Up to every 4 days or more**	4	8
**Bristol Classification**		
**Normal (3–4 or 5)**	36	72
**Constipated (1 or 2)**	13	26
**Diarrhea (6 or 7)**	1	2
**Constipation Score**		
**Normal (1–15)**	15	30
**Constipated (16–30)**	12	24
**Not apply**	23	46
**Wexner Classification**		
**Mild Incontinence (0–7)**	11	22
**Moderate Incontinence (8–13)**	3	6
**Severe Incontinence (13–20)**	7	14
**Not apply**	29	58

For continuous pressure variables, there was excellent agreement between C-ARM and HR-ARM. The ICCs for resting and squeeze pressures ranged from 0.88 to 0.92. This high level of agreement was consistent regardless of whether the functional anal canal was defined by the 50% pressure threshold (HR-ARM-FACL 50%) or the 10-mmHg pressure threshold (HR-ARM-FACL 10 mmHg). In stark contrast, the agreement for the functional anal canal length measurement itself was significantly lower. The ICCs were only 0.20 and 0.30 for the 50% and 10 mmHg definitions, respectively, indicating only slight to fair agreement (Table 4).

**Table 4:** Comparison of intraclass correlation coefficient levels, between conventional manometry and high-resolution anorectal manometry.

Parameter	ICC	IC 95%
**ARP – C-ARM x HR -ARM-FACL 50%**	0.89	0.81; 0.94
**ARP – C-ARM x HR-ARM-FACL 10 mmHg**	0.88	0.78; 0.93
**ASP – C-ARM x HR -ARM-FACL 50%**	0.92	0.86; 0.96
**ASP – C-ARM x HR-ARM-FACL 10 mmHg**	0.88	0.79; 0.93
**FACL – C-ARM x HR -ARM-FACL 50%**	0.20	−0.41; 0.55
**FACL – C-ARM x HR-ARM-FACL 10 mmHg**	0.30	−0.23; 0.60

ARP, anal resting pressure; ASP, anal squeeze pressure; FACL, functional anal canal length.

HR -ARM-FACL 50%: functional anal canal length as the length of the anal canal over which the pressures were greater than half the maximum resting pressure.

HR-ARM-FACL 10 mmHg: functional anal canal length as the region where pressures were at least 10 mmHg higher than the intra-rectal pressure.

Next, we assessed the diagnostic agreement for anal tone. This was done by categorizing the continuous resting and squeeze pressures as normal or abnormal based on the normative values for each method (Tables 1 and 2). Interestingly, the level of agreement was dependent on which HR-ARM definition of functional anal canal length (FACL) was applied. When the HR-ARM-FACL 50% definition was used, the agreement (K) for classifying resting and squeeze tone was fair and moderate, respectively. However, when the HR-ARM-FACL 10 mmHg definition was used, the agreement for both tones dropped to slight.

There was fair agreement between the two methods for the position of the high-pressure zone. In contrast, for the categorized functional anal canal length, the Kappa value was negative, indicating strong disagreement between C-ARM and HR-ARM (Table 5).

**Table 5:** Kappa levels of agreement between conventional manometry and high-resolution anorectal manometry according to normal values defined for each method.

Conventional anorectal manometry	High-resolution anorectal manometry				
Parameter	HR -ARM-FACL 50%		HR-ARM-FACL 10 mmHg		
	Kappa	IC 95%		Kappa	IC 95%
**Resting tone**	0.37	0.13; 0.61		0.18	0.05; 0.33
**Squeeze tone**	0.45	0.21; 0.69		0.18	0.05; 0.32
**Functional anal canal length**	−0.09	−0.24; 0.05		−0.13	−0.24; −0.03
**High-pressure zone position** ^*^	0.29	0.06; 0.52		0.29	0.06; 0.52

*Weight Kappa coefficient.

We then performed a sub-analysis to see if using a single reference standard would affect the diagnostic agreement. For this step, we categorized the pressure data from both C-ARM and HR-ARM using only the normative values established for C-ARM (Table 1). Under this condition, the agreement for classifying resting and squeeze tone was moderate when using the HR-ARM-FACL 50% definition, but fell to fair when using the HR-ARM-FACL 10 mmHg definition (Table 6).

**Table 6:** Kappa levels of agreement between conventional manometry and high-resolution anorectal manometry according to normal values defined for C-ARM.

	High-resolution anorectal manometry			
Parameter	HR -ARM-FACL 50%		HR-ARM-FACL 10 mmHg	
	Kappa	IC 95%	Kappa	IC 95%
**Resting tone**	0.8^*^	0.39; 0.78	0.31^#^	0.09; 0.53
**Squeeze tone**	0.45	0.21; 0.69	0.24	0.08; 0.41

HR -ARM-FACL 50%: functional anal canal length as the length of the anal canal over which the pressures were greater than half the maximum resting pressure.

HR-ARM-FACL 10 mmHg: functional anal canal length as the region where pressures were at least 10 mmHg higher than the intra-rectal pressure.

*Weight Kappa coefficient.

PS: For resting tone, hypertonic patients in C-ARM were excluded as in HR-ARM no patient received this classification.

**Fig 3:**
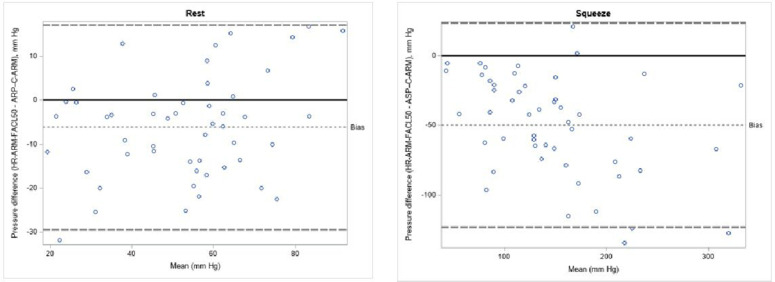
Magnitude of discordance between C-ARM and HR-ARM for analysis of the length of the anal canal over which the pressures were greater than half the maximum resting pressure. ARP-C-ARM: anal resting pressure. ASP-C-ARM: anal squeeze pressure. HR -ARM-FACL 50%: functional anal canal length as the length of the anal canal over which the pressures were greater than half the maximum resting pressure.

An analysis of the disagreement between the two methods was performed for the HR-ARM-FACL 50% definition (Fig 3). For resting pressure, HR-ARM systematically underestimated C-ARM values; the mean difference was −6.17 mmHg, with a 95% range for this difference from −9.50 to −2.85 mmHg. This underestimation was more pronounced for squeeze pressure, which showed a mean difference of −49.70 mmHg (95% range: −60.14 to −39.26 mmHg).

Furthermore, the graph for squeeze pressure revealed a proportional bias, as the magnitude of the difference between the methods grew larger at higher contraction pressures.

**Fig 4:**
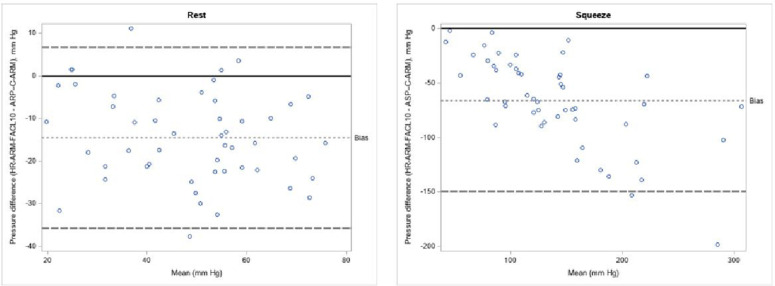
Magnitude of discordance between C-ARM and HR-ARM for analysis of the length of the anal canal by the region where pressures were at least 10 mmHg higher than the intra-rectal pressure. ARP-C-ARM: anal resting pressure. ASP-C-ARM: anal squeeze pressure. HR-ARM-FACL 10 mmHg: functional anal canal length as the region where pressures were at least 10 mmHg higher than the intra-rectal pressure.

A similar analysis was performed for the HR-ARM-FACL 10 mmHg definition (Fig 4). For anal resting pressure, this definition also resulted in a systematic underestimation of C-ARM values, with a mean difference of −14.49 mmHg and a 95% range for this difference from −17.51 to −11.47 mmHg. The underestimation for anal squeeze pressure was greater, showing a mean difference of −66.30 mmHg (95% range: −78.10 to −54.56 mmHg).

Consistent with the previous finding, the squeeze pressure graph also demonstrated a proportional bias, where the difference between the methods increased as the contraction pressures became higher.

When assessing signs of dyssynergic defecation, the level of agreement between the two methods varied by parameter (Table 7). Agreement was moderate for the overall visual signs, fair for incomplete rectal relaxion, but negative for intra-rectal propulsion, indicating disagreement.

**Table 7:** Kappa levels of agreement between conventional manometry and high-resolution anorectal manometry according to normal values defined for evaluation of dyschezia.

Conventional anorectal manometry	High-resolution anorectal manometry			
Parameter	HR -ARM-FACL 50%		HR-ARM-FACL 10 mmHg	
	Kappa	IC 95%	Kappa	IC 95%
**Rectal propulsion in simulated evacuation**	−0.30	−0.53; −0.06	−0.33	−0.57;-0.08
**Anal relaxation in simulated evacuation**	0.39	0.12; 0.66	0.36	0.09;0.63
**Visual dyschezia signs**	0.57	0.32; 0.81	0.57	0.32; 0.81

HR -ARM-FACL 50%: functional anal canal length as the length of the anal canal over which the pressures were greater than half the maximum resting pressure.

HR-ARM-FACL 10 mmHg: functional anal canal length as the region where pressures were at least 10 mmHg higher than the intra-rectal pressure.

## Discussion

Anorectal manometry (ARM) is generally considered an easy-to-perform method with high reproducibility and minimal risk of complications [14,15]. The newer high-resolution (HR-ARM) technique builds upon these strengths, offering superior spatial resolution due to its greater number of microtransducers [16] and a reduction in motion artifacts [17–18].

However, a key challenge in the field is the lack of consensus on normative values, with different professionals using varying reference ranges—an issue that has been exacerbated by the widespread adoption of high-resolution manometry.

A large-scale study by Carrington et al. [19], which included 107 institutions across 30 countries, found significant discrepancies in all aspects of ARM, including indications, equipment, technique, data acquisition, and analysis. The authors suggested that these inconsistencies likely impact clinical interpretation, data sharing between institutions, and multicenter research. A key finding was that clinicians using HR-ARM tend to place greater emphasis on qualitative descriptions of overall anorectal function, whereas those using C-ARM focus more on quantitative pressure measurements.

Numerous international studies have sought to define normative values for various populations using both water-perfused (WP) and solid-state (SS) devices [11,19–23]. However, the applicability of these international norms in Brazil is questionable. A key Brazilian study by Pinto et al. [24], for instance, prospectively evaluated 40 asymptomatic volunteers using an eight-channel conventional manometer. They found that the average pressure levels in this Brazilian cohort differed significantly from those reported in American studies [9]. Despite this discrepancy, these American studies are still routinely used as the de facto standard in Brazil and were therefore chosen as the reference for our analysis.

Another key Brazilian study by Viebig et al. [18] retrospectively evaluated 50 patients using the same HR-ARM equipment as in our study. The exclusion criteria for their cohort included a diagnosis of pelvic dyssynergia, a history of anal surgery, or prior anal injuries. The study reported normative values stratified by sex for several key parameters: Resting pressure: 79.8 ± 4.0 mmHg for women; 72.2 ± 3.0 mmHg for men. Squeeze pressure: 170.7 ± 8.0 mmHg for women; 229.5 ± 17.0 mmHg for men. Functional anal canal length: 3.0 ± 0.1 cm for women; 3.3 ± 0.1 cm for men. However, a direct comparison between our findings and these normative values was not appropriate, as our study was designed to include a symptomatic patient cohort with primary complaints of constipation or fecal incontinence.

Several authors have conducted comparative studies of different ARM methods. In a notable example, Simpson et al. [25] evaluated 11 patients with fecal incontinence and 10 healthy controls using five different methods in sequence: side-hole WP, end-hole WP, micro balloon, microtransducer, and portable Peritron. While the authors found a satisfactory correlation between the techniques and concluded that no single method was superior, they did highlight a specific drawback of WP systems. They noted that WP systems are more prone to artifacts—such as those from gas bubbles mixing with fluid or from water leaking onto the buttocks—which can inadvertently trigger reflex or voluntary contractions of the anal and gluteal muscles.

In a prospective study of 60 incontinent patients, Leo et al. [26] compared a portable air perfusion device with a 10-channel WP system and found a strong positive correlation between the two methods. They reported high correlation coefficients for both resting pressure (r = 0.84) and squeeze pressure (r = 0.97).

The literature is inconsistent regarding pressure differences between high-resolution SS and WP systems. While some studies reported no significant difference in resting pressures, others have found conflicting results pressures [25,27,28]. For example, a study by Jones et al. [29]evaluated 29 symptomatic patients with both methods. Although they found a good correlation between the techniques, they demonstrated that the SS system recorded significantly higher resting and squeeze pressures. The work of Jones et al. [29] is particularly relevant to our study. First, their cohort was similar to ours, comprising symptomatic patients with primary complaints of constipation or incontinence. Second, we followed their rationale for evaluating symptomatic patients rather than healthy volunteers, as this provides a wider and more clinically representative range of sphincter pressures for comparison.

In a study of 80 healthy volunteers, Gosling et al. [7] compared a conventional WP system with a high-resolution WP system. They found a strong correlation between the two for both resting pressure and contraction increment (ICC = 0.76 and 0.91, respectively). However, they also reported that the HR-WP system measured resting pressures that were 10% lower and contraction increments that were 27% lower than the conventional method. This finding is consistent with the results of our study. This outcome, however, contrasts with the findings of Jones et al. [29], who reported higher pressures with their HR system. A critical distinction is that the Jones et al. [29] study compared a WP system to a high-resolution SS system, suggesting that the catheter technology (WP vs. SS) is a key variable influencing the pressure results.

Rasijeff et al [30] also compared SS and WP systems in 60 healthy volunteers, finding no difference in resting pressures. However, consistent with the findings of Jones et al. [29], they reported that squeeze pressures were significantly higher with the SS method. The authors concluded that comparisons across studies are often confounded by variations in technique, equipment, and patient populations. Therefore, they emphasized the critical need for device-specific normative ranges, illustrating the point with a powerful clinical example. They explained that a single pressure measurement could be incorrectly classified as hypotonic if compared to SS-derived norms, potentially leading to inappropriate treatment. Conversely, that same pressure value might be correctly classified as normal against WP-derived norms. This accurate classification would then rightly prompt clinicians to investigate other potential causes for a patient’s symptoms, such as sensory deficits or issues with rectal emptying.

Our study confirmed that while the numerical measurements for resting and squeeze pressures show excellent agreement between C-ARM and HR-ARM manometry, this correlation did not translate to diagnostic agreement. When pressure values were categorized using the specific normative values for each method, the level of agreement significantly decreased. This drop in agreement was particularly pronounced when the HR-ARM functional anal canal was defined using the 10-mmHg pressure threshold compared to the 50% pressure threshold. Finally, a sub-analysis using only the C-ARM normative values as a single reference standard improved the agreement slightly, but it did not fully resolve the diagnostic discrepancies between the two methods.

In a study of 14 constipated patients, Kang et al. [27] compared conventional WP manometry with an HR-SS system. While they reported a strong correlation for resting and squeeze pressures, they also found a significant difference in the measurement of functional anal canal length. This discrepancy in functional anal canal length is consistent with our own findings. A likely explanation for this shared result is the occurrence of motion artifacts produced by the manual pull-through technique required in conventional WP manometry.

Manometric parameters during simulated defecation are influenced by more than just the type of equipment used; they also depend on variables such as intra-rectal balloon distension, body position, and patient effort [5]. Highlighting one such confounding factor, Sauter et al. [31] suggested that the “complete relaxation” of the anal sphincter often recorded by C-ARM is actually an artifact. They proposed that this artifact is caused by the movement of pressure sensors out of the anal canal during the patient’s abdominal straining.

Noelting et al. [32] reported that a negative rectoanal gradient during simulated defecation is not a reliable parameter for diagnosing defecatory disorders, and suggested that the balloon expulsion test is more useful for this evaluation. Furthermore, they found that up to 20% of asymptomatic individuals exhibit paradoxical contraction of the anal sphincter during the maneuver.

Similarly, a study of 110 asymptomatic volunteers by Li et al. [33] concluded that a negative rectoanal pressure gradient is not a reliable indicator of impaired evacuation. The authors attributed this finding to two main factors: First, the standard left lateral position used during manometry does not reflect the physiological seated position, in which rectal pressures are typically higher. Second, the pressure measured in an empty rectum during the test is likely lower than the pressure generated in a stool-filled rectum during true defecation.

In our study, the overall agreement for dyssynergia markers between C-ARM and HR-ARM was moderate. This result likely stems from a key methodological difference between how the two techniques assess simulated defecation. With the conventional method we used, only anal canal pressures (i.e., sphincter relaxation) were evaluated during the maneuver; information on intra-rectal pressure was not captured. In contrast, HR-ARM allows for the simultaneous evaluation of both rectal and anal pressures, providing a more complete picture of evacuation dynamics. This fundamental difference in capability likely explains our specific findings. For the measure of anorectal muscle relaxation, which both methods can assess to some degree, the agreement was fair. However, for intra-rectal propulsion, a parameter that only HR-ARM can truly measure, the comparison yielded a negative Kappa value, indicating disagreement.

The literature presents a mixed view on the clinical superiority of HR-SS manometry compared to WP systems for diagnosing defecatory disorders. On one hand, studies like Kang et al. [27] suggest that HR-SS offers an improved evaluation of anorectal disorders due to its superior spatiotemporal resolution. On the other hand, the findings of Jones et al. [29] complicate this picture. Their study found no significant correlation in anal canal relaxation pressures between WP and HR-SS systems during simulated defecation. More surprisingly, Jones et al. [29] also reported that the conventional WP device was actually more reliable for identifying patients with obstructed defecation caused by paradoxical puborectalis contraction.

Recently, Anefalos et al. [34] evaluated 50 healthy volunteers aged 59 or younger (25 men and 25 women) according to the London Protocol. The authors compared their findings, obtained using a 36-channel HR WP manometry system, with other studies on high-resolution perfusion systems and inferred a statistical trend indicating differences across all studied manometric parameters for all articles.

This study has several limitations. First, the study cohort consisted exclusively of symptomatic patients and did not include a control group of healthy volunteers. Second, the small number of male participants may limit the generalizability of the findings to men. We also recognized that a potential learning effect could create a bias, as patient performance can improve with instruction during the examination. To mitigate this order effect, we randomized the sequence in which each patient underwent the C-ARM and HR-ARM procedures.

## Conclusion

While our study found excellent agreement between C-ARM and HR-ARM for the quantitative measurement of resting and squeeze pressures, this correlation did not translate to diagnostic agreement. The process of categorizing pressures using non-specific, currently available normative values significantly reduced the level of agreement. This finding underscores the critical need to establish unique, device-specific reference ranges to ensure accurate clinical interpretation and guide appropriate treatment.

## Supporting information

S1 Appendix Complete date. (XLSX)
